# Health Tract No. 327

**Published:** 1868-06

**Authors:** 


HEALTH TRACT No. 327.

### THE PULSE.

Of a healthy grown person beats seventy times in a minute ; there may be good health, down to sixty ; but if the pulse always exceeds 70, there is disease ; the machine is working too fast ; is wearing itself out ; there is fever or inflammation somewhere, and the body is feeding on itself, as in consumption, where the pulse is always quick, that is over seventy, gradually increasing with decreasing chances of cure, until it reaches 110 or 120, when death comes before many days. When the pulse is all the time over 70 for months, and there is even a slight cough, the lungs are affected.

Every intelligent person owes it to himself to learn from his family physician how to ascertain the pulse in health ; then, by comparing it with what it is when ailing, he may have some idea of the urgency of his own case, and it will be an importrnt guide to the physician. Parents ought to know the healthy pulse of each child ; as, now and then, a person is born with a peculiarly slow or fast pulse, and the very case in hand may be that peculiarity. An infant's pulse is 130 ; a child's of seven years about 80 ; from 20 to 60 years, it is 70 beats a minute, declining to sixty, at fourscore.

There are pulses all over the body, but where there is only skin and bone, as at the temples, it is more easily felt ; the wrist is the most convenient point. The feebleness or strength of the beats is not material, being modified by the fingers, pressure. Comparative rapidity is the great point ; near death,. it is 140 and over. A healthy pulse imparts to the finger a feeling as of a woolen string ; in fever, it feels harder, like a silk thread ; if there is inflammation which is always dangerous, it beats fast, spiteful and hard, as if a fine wire was throbbing against the finger. When the pulse beats irregularly, as if it lost a beat, then hurried to make it up, there is something the matter with the heart. But how ever unnatural you may think the pulse is, do not worry about it, take nothing, do nothing except by the advice of an intelligent physician.

